# Effectiveness of an oral health intervention program for children with congenital heart defects

**DOI:** 10.1186/s12903-018-0495-5

**Published:** 2018-03-23

**Authors:** Tine B. Sivertsen, Anne N. Åstrøm, Gottfried Greve, Jörg Aßmus, Marit S. Skeie

**Affiliations:** 1 Department of Clinical Dentistry, University of Bergen, Årstadveien 19, NO-5020 Bergen, Norway; 2 Department of Clinical Science, University of Bergen, NO-5020 Bergen, Norway; 3 Department of Heart Disease, Haukeland University Hospital, NO-5021 Bergen, Norway; 4 Department of Pediatrics and Adolescents Medicine, Haukeland University Hospital, NO-5021 Bergen, Norway; 5 Centre for Clinical Research, Haukeland University Hospital, NO-5021 Bergen, Norway

**Keywords:** Congenital heart defect, Childhood oral health, Dental care for children, Special care for children

## Abstract

**Background:**

Children with congenital heart defects (CHD) are reported to have poorer oral health compared with healthy children. The aim of the present study was to evaluate the effectiveness of an intensive oral health care program among children with CHD followed from infancy to the age of 5 years, by comparing their oral health status at 5 years with a control group of children with CHD who had not received the program.

**Methods:**

In this longitudinal study, children in western Norway with a need for lifelong follow-up due to congenital heart defects were invited to participate (*n* = 119). Children born in 2008–2011 were offered an oral health intervention program from infancy to the age of 5 years. The outcome measures for evaluating the intervention were dental caries prevalence, dental erosion, plaque index and gingival bleeding index. The data of the intervention group were compared with cross sectional oral health data of 5 year old controls with CHD born 2005–2007 (already published).

**Results:**

Early oral health intervention did not affect the prevalence of caries (25.3% versus 25.4%) or dental erosion (22.2% versus 19.7%) of children with CHD assessed at 5 years. Children in the intervention group were less likely than those in the control group to present with both dental plaque and gingival bleeding at age 5 years. In spite of no difference in caries prevalence between the groups, caries affected children (d_1-5_mft) in the intervention group had fewer teeth affected by caries than children in the control group (*p* = 0.06). The care index was reported to be higher in the intervention group compared with the control group, implying that fewer children in the intervention group suffered from untreated dentine caries. Parents in the intervention group were more likely to brush their children’s teeth twice a day than parents of children in the control group.

**Conclusion:**

The oral health promotive program did not influence the prevalence of caries nor dental erosion. However, the findings indicated better oral hygiene, reduced gingival bleeding and less untreated dentine caries in the intervention compared with the control group.

**Trial registration:**

ClinicalTrials.gov NCT03311438. Registration date: October 17th 2017, retrospectively registered.

**Electronic supplementary material:**

The online version of this article (10.1186/s12903-018-0495-5) contains supplementary material, which is available to authorized users.

## Background

Children with congenital heart defects (CHD) are exposed to many oral health risk factors [1, 2]. With all other medical concerns for the parents, oral health is in many cases not given priority. Parental attention is focused on the chronic disease and not on oral health [3]. In a study performed in two London hospitals, dental attitudes, knowledge and health practices of parents of children with CHD were found to be inferior compared with parents of healthy children [4].

CHD is the most common congenital defect and defined as an abnormality in the structure of the heart or the major vessels. The defects can be categorised as mild, moderate or severe [5]. Children with moderate and severe CHD have oral health risk factors which are characteristic for the group. Feeding difficulty, frequent vomiting, malabsorption, and increased energy demands due to increased respiratory and heart work, may lead to frequent meals even during the night [6]. Some heart medicines, e.g. digoxin medication when available in a sucrose-based suspension as Lanoxin®, shows significant correlation with caries experience [7, 8]. It has been speculated that the systemic effect of CHD might induce developmental defects of the enamel (DDE), since ameloblasts are highly sensitive to changes in metabolic conditions [9]. Enamel hypoplasia in the primary dentition has also been reported to be more frequent in children with CHD compared with healthy controls [10]. Furthermore, enamel defects with rough, pitted or exposed dentine surfaces increase the susceptibility for caries [11]. Additionally, some of the heart medicines are acidic [7, 12] and will together with the vomiting disposition, enhance the dental erosion risk. Due to these challenges, children with CHD worldwide are reported to have poorer oral health compared with healthy children [7, 13], i.e. more often affected by carious lesions [7, 14], more often plaque and gingivitis [15]. Dental erosion in primary dentition among 5-year-old Norwegian children with CHD has also been reported [14]. Compared to reported erosion prevalence in 5–6 year-old Swedish healthy children [16], prevalence of severe and very severe erosion in the Norwegian study were slightly more prevalent.

Infective endocarditis (IE) is an infection of the heart’s internal surface (endocardium) including the heart valves. Despite improved management of IE, it is still associated with high mortality and severe complications [17]. Untreated oral diseases causing a risk of bacteraemia such as periodontitis [18] or caries with untreated dental decay have been documented [19] and are among the etiological factors for IE [20]. The outcome of sepsis of oral origin in children who are medically compromised may be fatal. In spite of this, not all parents of children with CHD are aware that poor oral health entails a risk of developing IE [21]. Recent guidelines for the prevention of IE have narrowed the indications for antibiotic prophylaxis to certain types of CHD. Instead they emphasise the importance of good oral health [20]. It is, therefore, important that children with CHD are offered preventive oral health programs with close follow-up starting from an early age. To our knowledge, there are no prospective studies evaluating the effectiveness of oral health intervention among young children with CHD.

Focusing on an intervention group of children with CHD from infancy to 5 years of age, this study evaluates the effectiveness of an intensive oral health care program by comparing the clinical oral health status at 5 years with a control group of children with CHD who, in line with the general child population, received regular follow-ups in the Public Dental Service (PDS) from 3 years of age.

It was hypothesised that children in the intervention group would have fewer oral health problems at 5 years of age than the control group.

## Methods

This intervention study took place during the period January 2010–December 2016 and was conducted as a collaboration with the Department of Clinical Dentistry, Paediatric Dentistry, University of Bergen (UIB), the Department of Pediatrics and Adolescents Medicine, Haukeland University Hospital, Bergen, and the PDS in three western Norwegian counties. The study was approved by the Regional Committee for Medical and Health Research Ethics (2009/2264). Additionally, written informed consent was obtained from parents of all the participants.

### Study design and participants

**Fig. 1:**
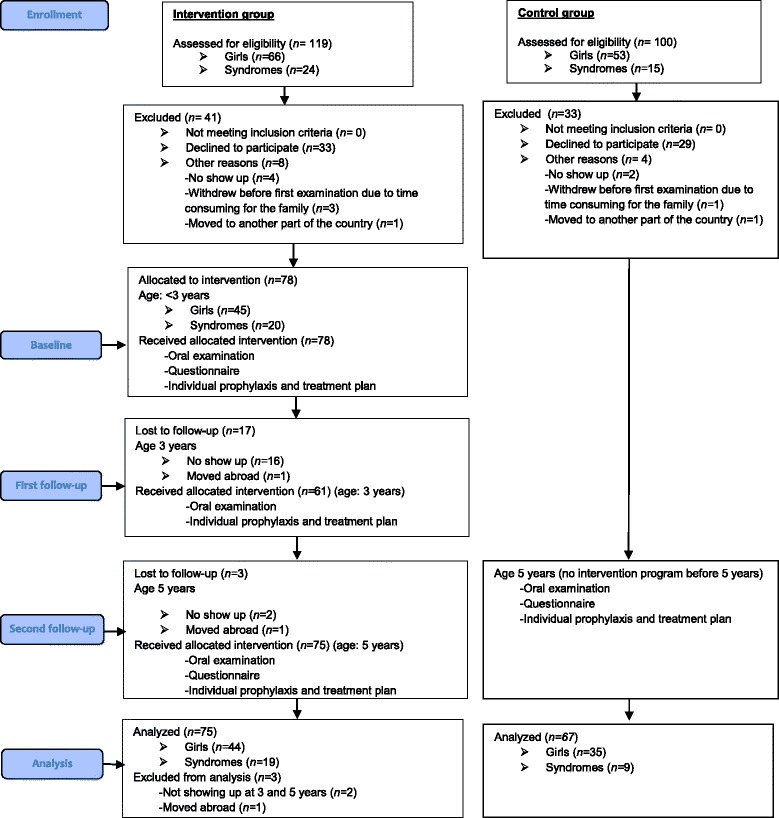
Flow chart of enrollment in intervention and control group of children with CHD

This was a prospective longitudinal design study, including four cohorts of children with CHD (born 2008–2011) who required lifelong follow-up for their CHD. The enrolment and follow-up of children with CHD in the intervention group is shown in Fig. 1. Parents, who did not respond to the initial letter of invitation for their child, received a reminder by mail 2 months later. All children in the intervention group were recruited before 3 years of age. A total of 119 children (66 girls) were invited to participate and receive an oral health intervention; at baseline (below 3 years), at first follow-up (3 years) and at second follow-up (5 years). Children included at baseline were considered lost to follow-up over time and excluded from final analysis if they withdrew from the study or did not meet at second follow-up. Because parents were responsible for their young children’s oral health, we did not exclude children with co-morbidities such as Down’s syndrome, Di George syndrome, Noonan syndrome, Cornelia de Lange syndrome, Williams’s syndrome and autism.

According to their CHD, the children were divided in three groups; *i)* left-to-right shunts, e.g. atrial septal defect and ventricular septal defect, *ii)* obstructions, e.g. coarctation of the aorta, aortic stenosis, and pulmonary stenosis, and *iii)* complex heart defects, e.g. tetralogy of Fallots, transposition of the great arteries and hypoplastic left or right heart syndromes. At 5 years, data on oral health and oral health related background factors were compared with corresponding published data of 5-year-old children with CHD, also from western Norway (*n* = 67, born 2005–2007). The latter group served as controls [14]. These children were recruited at the age of 5 years and had not been offered the intensive oral health intervention program received by children in the intervention group (Fig. 1.) The control group had been followed in the PDS in line with the general population of children, who are offered the first oral examination from 3 years of age.

The caries prevalence (at dentine level) of 5 year olds in the control group had previously been compared with the corresponding prevalence of 5 year olds in the general population from the national health register “KOSTRA” (Municipality-State-Report of 2010, 2011 and 2012 in three counties in western Norway, *n =* 18,974) [14]. Likewise the caries prevalence at age 5 years of the present intervention group were compared with the general population (Municipality-State-Report of 2013, 2014, 2015, 2016, *n =* 26,161) [22]. Possible time trends in caries epidemiology of 5 year olds during 2010–2016 were also assessed.

### Children’s clinical oral examinations and clinical outcome measures

At baseline (below 3 years of age) a simplified oral examination was performed. Children were examined sitting on a parent’s lap in the dental chair or on a regular chair examined by the “lap to lap” method. At the first follow-up examination at 3 years of age, the child sat either on parent’s lap or alone in the dental chair. Procedures for the oral health examination of the intervention group at 3 and 5 years of age and of the control group at 5 years of age were the same. Examinations were performed using a mouth mirror and a dental probe under good lighting conditions. Caries and DDE were registered for all dental surfaces while index teeth were used for dental erosion, dental plaque and gingival bleeding. Bitewing radiographs (BW) were only taken in 5 year olds, when there were molar contacts. Parents with immigrant background were offered interpreting services if desired. All oral health examinations were conducted by the same two dentists who previously had collected the published inter-observer (Cohen’s kappa value for caries 0.77 and 0.86) and intra-observer (Cohen’s kappa value for caries 0.85) reliability data of the control group [14].

Outcome measures for evaluating the effectiveness of the intervention program, assessed at age 5 years, were dental caries, dental erosion, plaque index (PI) and gingival bleeding index (GBI). DDE was not an outcome measure of the intervention program as this disturbance occurs during tooth mineralisation. As DDE sometimes lead to exposed dentin through post-eruptive breakdown, it was seen upon as important to include DDE in the description oral health, especially among children with CHD who should avoid dentine exposure. DDE was also assessed to see if the prevalence in the intervention and the control group were similar and to compare with corresponding prevalence from healthy children, reported in literature. Criteria for the various diagnostic systems were as follows: For caries, a five-graded diagnostic system was used [23], where enamel lesions were characterised as grades 1 or 2, and dentine lesions as grades 3, 4, or 5. Secondary caries, filled and extracted (due to caries only) teeth and the care index (a fraction with filled teeth as numerator and d_3–5_mft count as denominator) [24]. For dental erosion, a four graded diagnostic system [16, 25] was applied on buccal and palatal surfaces of the primary maxillary anterior teeth (53–63) and the occlusal surfaces of all primary molars. Erosion grade 3 was considered severe and grade 4 very severe. A modified DDE index [26] was selected for DDE registration in which demarcated opacities were coded 1, diffuse opacities 2, demarcated and diffuse opacities 3, hypoplasia 4, hypoplasia and opacities 5, post-eruptive breakdown of enamel 6, and atypical fillings 7. Finally, for dental plaque and gingival bleeding, the PI [27] and GBI [28] were reported around the marker teeth 55, 51, 65, 75, 71, and 85. The presence of plaque in the four amelo–gingival areas was assessed with a dental probe and absence of debris was recorded as score 0 and presence as score 1. Gingival bleeding was recorded by probing cautiously using a WHO periodontal probe with a 0.5-mm ball tip on six surfaces; mesio-buccal, in the middle of the buccal surface, disto-buccal, mesio-lingual, in the middle of the lingual surface and, disto-lingual. If bleeding occurred within 10 s in at least one of the places, the tooth was scored 1 (bleeding), otherwise the tooth was scored 0.

Dichotomised outcome variables for the various clinical outcome measures were constructed for use in logistic regression models; presence (1) or absence (0) of caries into dentine, or missing or filled teeth, of erosion close to or into the dentine (grade 3–4), of plaque on at least one of index teeth, and of gingival bleeding around one of the index teeth.

### Parental questionnaire and anamnestic factors

Parents of children in the intervention group were asked to respond to a questionnaire at baseline and at the second follow-up, while parents in the control group were asked at examination at age 5 years. The questionnaire contained 30 structured items, the same questionnaire as used in the Norwegian article previously referred to [14]. As to the validation described in this article, a prior pilot study had at that time been conducted and most of the items were taken from other validated questionnaires [29–31]. The topics covered were socio-demographic and anamnestic items, items about parental oral health attitudes, oral health related behaviours, and oral health promotion. The original and recoded response scales for the questionnaire data are shown in Table 1. The anamnestic background variables were coded cyanotic (1), not cyanotic (0), heart failure (1) and heart failure not present (0), taking heart medication (1) and does not take heart medication (0), low birth weight < 2500 g (1) and normal birth weight > 2500 g (0).

**Table 1:** Items from the questionnaire with original coding and new categories with recoding

	Original coding						Recoding	
Item	Code 1	Code 2	Code 3	Code 4	Code 5	New category	Code 0	Code 1
Place of birth of the mother and place of birth of the father	Born in Norway	Born elsewhere	Open category; which country?			Parents origin	Both Scandinavian	At least one of the parent’s origin as non-Scandinavian
Mother`s education and father`s education	Primary or secondary school	Vocational school	College or university			Parents total education	High education (at least one of the parents with original code 3)	Low education (both parents original code 1 or 2)
“How old was your child when you started brushing his/her teeth?”	Under 1 year	Between 1 and 2 years	More than 2 years			Start age of tooth-brushing	Before 1 year of age	After or at 1 year of age
“How often is the child’s teeth brushed?”	Never	Most days	Once a day	Twice or more a day		Brushing habits	Twice a day or more	Less than twice a day
“Does the child use fluoride tablets?”	Yes	No				Use of fluoride tablets	Yes	No
“How often does your child eat sugary snacks and drinks between meals?”	Every day	Most days	Once a week	Sometimes	Never	Intake of sugary snacks and drinks between meals	Once a week or less	More than once a week
“Was the child bottle fed with milk or juice after 1 year of age?”	Yes	No				Bottle feeding with milk or juice > 1 year of age	No	Yes
“Does the child have drink or food in bed before sleeping or during the night?”	Yes	No				Food or drink in bed before sleeping or during the night	No	Yes
“Has the child had sugar water intake at the hospital as a consequence of CHD?”	Yes	No				Sugar water intake at the hospital as a consequence of CHD	No	Yes
“How would you rate the dental health of your child?”	Very good	Good	Neither good nor bad	Bad	Very bad	Parental rating of child’s dental health	Good (original code 1 and 2)	Bad (original code 3, 4 and 5)

### Intervention

At baseline (before age 3 years) all parents in the intervention group received a standardised “knowledge package”, included “Lift the lip program” [32] compiled in Table 2. Both general and local interventions were repeated at first follow-up at 3 years and second follow-up at 5 years. The oral health risk assessment was based on and adjusted to previous articles in caries risk assessment available before the onset of the study [33, 34]. Risk assessment was made by the following background factors: 1) dental factors such as visible caries, visible plaque, gingivitis, erosion and DDE; 2) parental oral health related behaviours such as oral hygiene habits, dietary habits and use of fluoride; 3) social factors such as immigrant background where parents showed poor language skills, and 4) medical factors such as comorbidity and use of medication with drugs sweetened with sucrose.

**Table 2:** The preventive oral health intervention program

The content of the intervention program performed in intervention group at baseline, first – and second follow-up		
Preventive oral health intervention program		
General intervention		
*A knowledge base*	Importance of optimal oral hygiene	-Explaining the link to IE
	Other counselling	-Medicine intake: before brushing teeth -If drinking during night, only water
	“Lift the lip” program	-To detect early caries lesions and how to intervene - contact PDS
*Motivation*	Promotion of parental discussions	-Explaining the benefits of optimal oral health
*Behavioural change intervention.*	Oral hygiene instruction	-Supervised tooth-brushing twice daily (fluoride paste ≥1000 ppm) -Instruction of tooth-brushing technique
	Dietary counselling	-Reduced intake of sugary snacks and sugary or acidic drinks
Local intervention		
*Fluoride supplements*	Fluoride tablets not a local intervention	-Dose according to age
	Professional varnish application (Duraphat ®)	-In moderate amounts, only on caries risk surfaces
Programs according to oral health risk assessment. Recommendation for PDS		
Program I (Pro. I)	Appointment regime	-Twice annually Go through extradited knowledge pack.
	Program	-Practical training of the parents in how to brush their child’s teeth -Fluoride tablets -Duraphat®
Program II (Pro. II)	Appointment regime	-Four times annually
	Program	-Pro I and additional: -Information about location of enamel caries, erosion or DDE -For immigrants, clarify that the message on oral health were understood. If not, interpreter service offered
Program III (Pro. III)	Appointment regime	-Initially every month for 3 months -Depending on the response, maintain same appointment regime or change to four times or twice annually
	Program	-Pro I + II and additional: -Rapid onset of acute treatment -Additionally Duraphat® every month for three months, then once during the remaining year

The recommendation from the caries risk assessment was the most intense preventive program for children with the highest caries risk (those children with the highest number of caries risk determinants). The children were evaluated based on data from the oral examination, anamnestic information and information obtained from the questionnaire and accordingly assigned to different promotive oral health intervention programs, denoted as Program I, II and III (Table 2). As all the participants had CHD, stated previously as a risk for reduced oral health [14], the most modest program applied was Program I. The children offered this program had no other known risk factors for poor oral health than CHD. Program II was assigned to children who had association with other known risk groups in terms of poor oral health or had signs of oral disease such as a) enamel caries grade1–2, erosion or DDE; b) findings from the questionnaire corresponding to additional risk for oral disease beyond what is normal for the group; c) immigrant background where parents show poor language skills and simultaneously caries lesions and; d) co-morbidities with additional risk for oral disease. Program III was assigned to children who also had other known risk determinants like very poor oral health, signs of severe oral disease or where findings from the questionnaire corresponded to serious risk of oral disease. After categorising the children to the various programs, each child’s responsible dentist or dental hygienist at the local PDS clinic was contacted by telephone with information about the project and the findings of the examination. A written report with a treatment (non-operative and operative) plan and recommendation for follow-up was sent after each oral examination: at baseline, first follow-up and second follow-up. The children were offered all necessary dental treatment in the PDS.

The initial intervention plan for recommendations for the PDS dentists for follow-up of the highest risk children (Program III) included an option of recalls every month during a 3 month period. Compliance by the PDS staff with the recommendation for follow-up was evaluated when the second follow-up for all children was completed. The child’s local PDS-clinic was contacted and feedback in the form of a written list of the child’s appointments in the PDS in the period from baseline to the second follow-up was received. There was no information about clinical procedures undertaken, only dates of appointments.

### Statistical methods

Descriptive statistics were used to characterise the intervention and the control group. The effectiveness of an early oral health intervention was estimated by comparing intervention and control groups on clinical oral health outcomes at age 5 years using a multiple logistic regression model for each outcome variable. The model was fitted unadjusted and adjusted for one of the following independent variables at a time: brushing habit, start age of tooth- brushing, diet habit, parent’s origin, parent’s education, bottle-feeding, night meals, sugar water, sex, heart problems, cyanosis, birth weight, heart medication and syndrome. The various dependent dichotomised outcome variables for evaluating the effectiveness of the intervention program were caries experience, dental erosion, PI and GBI. The independent variables used in the logistic regression were selected in relation to known caries risk determinants and demographic background factors, which could influence the oral outcomes. In the final model, maximally adjustments were included to guarantee sufficient power. The adjustments with the largest deviation from odds ratio (OR) were selected in the unadjusted model if the deviation was at least 10% of the unadjusted OR. For other comparisons chi-square and independent sample *t*-test were used. The general significance level was set to 0.05. The Bonferroni correction was used to address multiple comparisons leading to the marginal significance levels of 0.0028 when comparing characteristics between the intervention and control group, and 0.01 for the logistic models. All analyses were performed using SPSS version 23 (Inc. Chicago, IL, USA) and Matlab 9.0 (Mathworks Inc.).

## Results

The response rate for the intervention group at baseline (mean age: 22.2 mon, SD 8.41, range 6 to 35 mon) was 65.5% (78/119) and at 5 years (mean age: 62.7 mon, SD 3.96, range 55 to 76 mon) it was 63.0% (75/119). Due to lack of time by the parents, seventeen (17/78) children did not attend for the first follow-up at 3 years, but fourteen of them (14/17) did however come for the second follow-up at 5 years. Mean age at first follow-up was 39.6 mon (SD 4.6, range 28 to 51 mon). The response rate for the control group was 67%. BW radiographs were taken of 57.3% of the children in the intervention group and of 58.2% in the control group. The reasons for omission were mostly lack of cooperation or gag reflex, but lack of inter-molar contact was also reported.

### Comparisons of the intervention and control group

Social, medical, behavioural and dental characteristics of children in the intervention and the control group at age 5 years are shown in Table 3. The groups differed significantly only with respect to use of heart medication, which was significantly more frequent in the control than in the intervention group (*p* < 0.001). Tooth-brushing habits were also reported to be more favourable in the intervention than in the control group (less than twice a day tooth-brushing: 25.3% vs. 50.7%, *p* = 0.004). Sugar water given at the hospital during examination was reported to be more common in the intervention compared with the control group (49.3% vs.25.4%, *p* = 0.006). As shown in Table 4, the prevalence of caries into dentine, and missing or filled teeth (d_3–5_mft) did not differ between the intervention and control group (25.3% versus 25.4%) neither in unadjusted nor in adjusted logistic regression analyses (*p* = 0.878). Dmft values in both groups were calculated and even though no differences in the proportions of children with caries in the two groups were found, among those with caries experience, the mean d_1-5_mft-value was 4.03 (SD: 3.16) in the intervention group compared with 5.96 (SD: 4.21) in the control group (*p* = 0.06). None of the children in the intervention group had lost teeth due to caries compared with 3% in the control group. A higher proportion of children in the intervention than in the control group presented with filled teeth due to caries (14.7% versus 6.0%).The care index in the intervention group was 38.5% versus 10% in the control group.

**Table 3:** Social, medical, behavioural and dental characteristics of children with CHD at 5 years of age in the intervention- and in the control group

Sample characteristics	Intervention group (*n* = 75)		Control group (*n* = 67)		
	Valid observations	N (%)	Valid observations	N (%)	*p* value
Age^a^	75	5.23 (0.33)	67	5.25 (0.34)	0.710
Girls	75	44 (58.7%)	67	35 (52.2%)	0.442
Syndrome	75	19 (25.3%)	67	9 (13.4%)	0.075
Heart defects	75		67		0.039
Left to right shunts		27 (36.0%)		15 (22.4%)	
Obstructions		17 (22.7%)		10 (14.9%)	
Complex		31 (41.3%)		42 (62.7%)	
Cyanotic heart defect	75	29 (38.7%)	67	28 (41.8%)	0.705
Use of heart medication	75	17 (22.7%)	67	39 (58.2%)	< 0.001
Experienced heart failure	75	41 (54.7%)	67	35 (52.2%)	0.772
Low birth weight < 2500 g	75	15 (20%)	61	7 (10.4%)	0.179
At least one parent with non-Scandinavian origin	75	10 (13.3%)	67	6 (9.0%)	0.410
Parents total education low	75	27 (36.0%)	67	19 (28.4%)	0.331
Start age tooth-brushing ≥1 year of age	75	14 (18.7%)	66	18 (26.9%)	0.223
Tooth-brushing < twice a day	69	19 (25.3%)	66	34 (50.7%)	0.004
Intake of sugary snack and drink between meals > once a week	69	9 (12%)	66	11(16.4%)	0.579
Bottle feeding with milk or juice > 1 year of age	75	44 (58.7%)	67	32 (47.8%)	0.193
Food or drink in bed before sleeping or during the night	69	26 (34.7%)	66	18 (26.9%)	0.197
Use of fluoride tablets	74	54 (73.0%)	64	52 (81.3%)	0.251
Sugar water intake as a consequence of CHD	74	37 (49.3%)	63	17 (25.4%)	0.006
Parenteral rating of child’s dental health as bad	70	13 (18.6%)	67	6 (9.0%)	0.104
Developmental defect of the enamel	73	29 (39.7%)	60	17 (28.3%)	0.169

^a^Mean (SD), independent t-test

*p* < 0.0028

**Table 4:** The prevalence of oral conditions in the intervention and the control group at 5 years of age. Odds ratio (OR) and 95% confidence interval (CI) and *p*-values for unadjusted and final logistic regression model for having oral conditions in the intervention compared to the control group

	Intervention group (*n* = 75)		Control group (*n* = 67)		Unadjusted model		Final model	
	Valid observations	N (%)	Valid observations	N (%)	OR (CI)	*p-value*	OR (CI)	*p-*value
Caries tooth level								
Caries into dentine, missed or filled (d_3–5_mf)	75	19 (25.3)	67	17 (25.4)	0.998 (0.468,2.128)	0.996	1.067 (0.466,2.445)	0.878^a^
Enamel caries (d_1–2_)	75	21 (28.0)	67	19 (28.4)				
Dentine caries (d_3–5_)	75	14 (18.7)	67	16 (23.9)				
Missed due to caries	75	0 (0.0)	67	2 (3.0)				
Filled	75	11 (14.7)	67	4 (6.0)				
d_1-5_mf	75	29 (38.7)	67	25 (37.3)				
Erosion close to or into the dentine (grade 3–4)	72	16 (22.2)	61	12 (19.7)	1.167 (0.503,2.705)	0.719	1.120 (0.417,3.013)	0.822^b^
Erosion total (grade 1–4)	72	54 (75.0)	61	34 (55.7)				
Plaque on at least one of index teeth	73	43 (58.9)	66	59 (89.4)	0.170 (0.068,0.423)	< 0.001	0.173 (0.064,0.468)	0.001^c^
Gingival bleeding around one of index teeth	71	3 (4.2)	61	13 (21.3)	0.163 (0.044,0.603)	0.007	0.152 (0.038,0.604)	0.007^d^

Adjusted for: ^a^ brushing habit and sugar water, ^b^ brushing habit and heart medication, ^c^ brushing habit and diet habit, ^d^ diet habit and parents origin

*p* < 0.01

As shown in Table 4, the prevalence of erosion close to or into the dentine (grade 3 and 4) in the intervention and the control group was respectively 22.2% and 19.7%. In both groups, the palatal surfaces of primary maxillary incisors were the most affected surfaces. The groups did not differ significantly regarding dental erosion in the final multiple variable logistic regression models (*p* = 0.822). The proportion with plaque in at least one index tooth amounted to 58.9% and 89.4% in the intervention and control group, respectively. Adjusting for background variables in the logistic regression model revealed that the odds ratio for having dental plaque in the intervention compared with the control group was 0.173 (95% CI 0.064–0.468). Moreover, children in the intervention group were less likely than children in the control group to present with gingival bleeding: OR 0.152 (95% CI 0.038–0.604). Results for all logistic regression models are shown in supplemental tables (Additional file 1: Table S1, Additional file 2: Table S2, Additional file 3: Table S3 and Additional file 4: Table S4).

### Comparisons with the general population of 5-year-old children

**Fig. 2:**
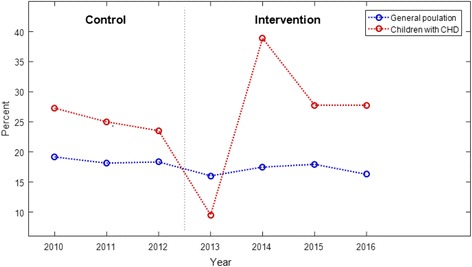
The red line illustrates the caries prevalence (d_3–5_mft) among 5-year-olds with CHD (the control group from 2010 to 2012 and intervention group from 2013 to 2016. The perimeter marked by a dotted line) and the blue line the general population of 5-year-olds in the selected counties in western Norway from 2010 to 2016

Figure 2 illustrates that the dentine caries prevalence during year 2010–2016 in children with CHD in the control and intervention group respectively, was on average higher compared with the general population of 5-year-old children in western Norway, except for the year 2013.

### Follow-up of the intervention group in the PDS

Table 5 shows that 72% (54/75) of the children were not followed in the PDS according to written recommendations for follow-up given in letters. Mean missed number of follow-up appointments in the PDS was 3.78, with a range from 1 to14. There was no significant difference in the outcome oral health variables (caries (d_3–5_mft), erosion into or close to the dentine (grade 3–4), plaque on at least one of the index teeth, and gingival bleeding around one of index teeth) at 5 years old in the intervention group (*n* = 21), followed-up according to the PDS recommendation for follow-up compared with those who were not (*n* = 54) (Table 5).

**Table 5:** Compliance according to the recommendation for follow-up interval in the PDS in the time gap from baseline (mean age 2 years) to the second follow-up at 5 years and the outcome data for the intervention group at 5 years for dentine caries, erosion into or close to the dentine, PI, and GBI

	Advice for follow-up followed		Advice for follow-up not followed		
	Total	N (%)	Total	N (%)	*p*-value
Caries into dentine, missed or filled teeth (d_3–5_mft)	21	6 (28.6%)	54	13 (24.1%)	0.688
Erosion into or close to the dentine (grade 3–4)	20	3 (15.0%)	52	13 (25.0%)	0.361
Plaque on at least one of index teeth	19	9 (47.4%)	45	19 (42.2%)	0.705
Gingival bleeding around one of index teeth	19	1 (5.3%)	44	2 (4.5%)	0.902

*p* < 0.05

Among children with initial or manifest caries at baseline before 3 years of age or first follow-up at 3 years of age (8/75), four of the eight children were not followed-up in the PDS as recommended. All of them had dentine caries or fillings at second follow-up at 5 years. Among those (4/8) who were followed-up according to the recommendations, two had and two had not dentine caries or fillings at the second follow-up at 5 years.

## Discussion

To our knowledge, this study is the first study to evaluate an early oral health intervention program implemented among children with CHD between the ages of two and 5 years. According to the present findings of this study, an early oral health intervention did not affect the prevalence of caries nor of dental erosion in children with CHD, assessed at 5 years of age. Plaque on at least one index tooth was noted both in the intervention and in the control group. However, children in the intervention group were significantly less likely than those in the control group to present with both dental plaque and gingival bleeding at age 5 years in unadjusted and adjusted analyses. In spite of no difference in caries prevalence between the two groups, caries-affected children (d_1-5_mft) in the intervention group had fewer teeth with caries experience than their counterparts in the control group (*p* = 0.06). The care index was also reported to be much higher in the intervention group compared with the control group, implying that fewer children in the intervention group suffered from untreated dentine caries. Moreover, parents in the intervention group were more likely to brush their children’s teeth twice a day than parents of children in the control group. The present study partly confirms the hypothesis that children in the intervention group would present with fewer oral problems than children in the control group.

Caries is a preventable disease and in the light of the intensive and individualised preventive advice received by the intervention group, the expectation of a lower number of individuals with caries in the intervention compared with the control group was not attained. Poor responsiveness to dental preventive efforts among young children with CHD has been reported by other researchers. A study in Sweden showed that in spite of intensive preventive efforts in children with CHD, the cardiac group had more caries in the primary dentition compared with healthy controls, especially children on digoxin medication [7]. For the present study, some possible reasons for the absence of preventive effect on caries prevalence among the children should be considered. Compared with the controls, better oral hygiene and more frequent tooth-brushing occurred in the intervention group at age 5 years. In spite of the absence of an intervention effect on clinically assessed caries experience, improved tooth-brushing might have occurred as a result of the intensive oral health care intervention. This accord with findings reported by a study in Scotland where improvement in five-year-olds dental health was detected after introduction of a national supervised tooth-brushing program [35]. Parental monitoring of children’s brushing behaviour is complex and it takes time both to encourage positive oral health attitudes and to transform the attitudes about brushing to favourable routines [36]. It has been shown that young children (2.5- to 3.5 years old) with initial carious lesions, have a high risk of caries progression and the development of new carious lesions [37], implying that caries prevention should start at an early age, preferably before the disease has reached dentine. The compliance of PDS staff with recommendations for follow-up interval showed, when evaluated, that only half of the group with enamel or dentine caries at baseline before 3 years or first follow-up at 3 years were followed-up as recommended; this probably had a negative effect on the outcome. Additionally, the parents, collaboration was not always optimal as seventeen children did not attend the first follow-up due to the parents’ lack of time. This only underlines that although the principles of caries prevention are simple, the implementation, management and evaluation of programs and recommendations can be difficult [38]. It may also be that oral health interventions are not 100% effective and that CHD per se leads to poor oral health due to many oral health risk factors [7].

Compared with the intervention group, the use of heart medication was more frequent in the control group. This may indicate more severe heart disease in the control group compared with the intervention group. Differences between the groups in relation to use of heart medication could have influenced caries occurrence, as paediatric heart medication has been shown to entail a higher caries risk [7]. In case of digoxin medication, the longer period of medication, the higher dmfs-value was found [7]. However in the present study an association between heart medication and caries was not found in the control group [14]. On the other hand, higher consumption of sugar water in the intervention group was reported and the role of sugar in caries initiation and development is for long time established [39]. A study has also documented that babies who were routinely fed sweetened water during the first month of life had greater preference for sweetened water at 6 months and at 2 years of age, compared with children who did not consume sweetened water as infants [40].

Children in the control and intervention group were born in different periods of time, the control group between 2005 and 2007 and the intervention group between 2008 and 2011. However, during these years, dentine caries prevalence in the general population of five-year-old children in western Norway was shown to be stable [22]. A common trait of the present study was that the caries prevalence in children with CHD at 5 years, in both the control and intervention group, was on average higher than in the general population, except for children who met to follow-up in 2013 who had lower caries prevalence. A possible explanation for the lower caries prevalence is a small sample size of the group.

Early information to parents of the intervention group regarding risk factors for dental erosion such as vomiting, acidic food, beverages and medicines was provided, however, no difference in dental erosion close to or into the dentine between the intervention and the control group was seen (22.2% vs. 19.7%). Prevention of dental erosion can be difficult, especially in the primary dentition, since children with CHD are often prone to frequent vomiting in the first years of life and have an intake of acidic medicines [7, 41]. Information alone does not interfere on the vomiting frequency which is beyond the control of the patient, and the medicines ordered by doctors will be taken, acidic or not. The finding of better oral hygiene among the intervention group, though being promising as regard to future caries development, it might not have the same impact on erosion as plaque is seen to act protective for erosion development [42]. Studies from Norway have also suggested that the susceptibility to dental erosion is influenced by genetic variation and that, for certain individuals, only minimal acidic exposure may be sufficient to cause damage to the teeth, while others may never develop dental erosions despite extensive exposure to acid [43, 44].

The prevalence of DDE in the intervention and the control group was expected to be at the same level, since DDE will occur before eruption of teeth and the medical characteristics of the groups did not differ significantly. The respective prevalences in the intervention and control group (39.7% vs. 28.3%) were higher than that reported in a corresponding study among healthy German children with a DDE prevalence of 5.3% [45]. Cyanosis and heart failure, resulting in reduced oxygen to peripheral tissues, could interfere and disturb dental development. Even though in recent years there has been a decline in operation age and results of heart surgery are better, also for severe heart defects [46], developmental disturbances in primary teeth could potentially occur even before the birth of the child. This could be a possible explanation for a higher prevalence of DDE in the primary dentition among children with CHD.

According to the burden of different oral health problems revealed and to the parental rating of their child’s dental health, it should be suggested that some children also suffered from reduced oral health related quality of life [47].

A major strength of the present study was the longitudinal design. Oral health promotive studies with a longitudinal design are required, especially when the baseline is early age, as in the present study with an average age at baseline at 22 months. Further strengths of the present study were a low drop-out rate from baseline to second follow-up at 5 years of age and that only two experienced dentist conducted all the examinations; they had good to excellent agreement [14]. As the referring hospital treated all children with CHD in western Norway, the study was representative of the region, but probably not for the entire population of children with CHD in Norway. Children with syndromes were not excluded because parents are responsible for their young children’s oral health. Though some syndromes are considered to have various oral health challenges, the literature is inconclusive regarding dental caries, oral hygiene and gingival health in this group of children [48, 49]. No significant difference according to caries, erosion and DDE was found between children with CHD and children with CHD and additional syndromes in a previous Norwegian study [14].

One limitation was that the age at the baseline examination in the intervention group varied between 6 months and 35 months. The main reason for the variation at baseline was the practical problems for the parents who had to manage multiple visits to the hospital for the heart disease, medical treatment and other examinations. The fact that we had no direct control over the follow-up intervals nor the treatment done in the PDS was also a limitation. The outcome of the promotive oral health intervention program was probably negatively affected by the fact that 72% of the children were not followed-up at their local PDS clinics and that the recommendations for follow-up provided at baseline and the first follow-up were not implemented. It has been shown that parents of children with CHD are less satisfied with the reception at the dental clinic than parents of children with healthy children [50], suggesting that children with severe CHD should receive dental care in clinics for paediatric dentistry, especially at early ages. The first 2 years have actually been suggested to be the most important period for succeeding with oral health effective interventions [51]. The association between oral and general health is well documented, but for children with CHD it should be considered essential.

## Conclusions

This intensive oral health promotion program did not influence the caries or the dental erosion prevalence since no differences between the intervention and control group occurred in caries or dental erosion prevalence at the age of 5 years. However, the findings indicated better oral hygiene, reduced gingival bleeding and less untreated dentine caries in the intervention compared to the control group, all beneficial for avoiding future risk of infective endocarditis.

## Additional files

Additional file 1: **Table S1.** containing the outcome variable caries and independent background factors for logistic regression model comparing the intervention with the control group. (DOCX 16 kb)

Additional file 2: **Table S2.** containing the outcome variable dental erosion and independent background factors for logistic regression model comparing the intervention with the control group. (DOCX 16 kb)

Additional file 3: **Table S3.** with outcome variable dental plaque and independent background factors for logistic regression model comparing the intervention with the control group. (DOCX 15 kb)

Additional file 4: **Table S4.** with outcome variable gingival bleeding and independent background factors for logistic regression model comparing the intervention with the control group. (DOCX 15 kb)

## Abbreviations

ANÅ: Anne Nordrehaug Åstrøm
BW: Bitewing radiographs
CHD: Congenital heart defects
CI: Confidence interval
DDE: Developmental defects of the enamel
Dmft: Decayed, missed, filled teeth
GBI: Gingival bleeding index
GG: Gottfried Greve
IE: Infective endocarditis
JA: Jörg Aßmus
MMS: Marit Slåttelid Skeie
OR: Odds ratio
PDS: Public dental service
PI: Plaque index
SD: Standard deviation
SPSS: Statistical package for the social science
TBS: Tine Birkeland Sivertsen
