# Supplementary material for: Effectiveness of an oral health intervention program for children with congenital heart defects
Source: BMC Oral Health. 2018 Mar 23;18:50. doi: 10.1186/s12903-018-0495-5 (PMC5865357; doi:10.1186/s12903-018-0495-5)
Supplement: Supplementary file 1 — Table S1. containing the outcome variable caries and independent background factors for logistic regression model comparing the intervention with the control group. (DOCX 16 kb) [file 12903_2018_495_MOESM1_ESM.docx]

Table S1: Odds ratio (OR) and 95% confidence interval (CI) and p-values for outcome variable caries and independent background factors for logistic regression model in the intervention compared to the control group.

|  | N | OR | CI | *p-value* |
| --- | --- | --- | --- | --- |
| Unadjusted | 142 | 0.998 | (0.468,2.128) | 0.996 |
| Brushing habit | 135 | 1.266 | (0.569,2,816) | 0.563 |
| Start age of tooth-brushing | 141 | 1.011 | (0.471,2.173) | 0.977 |
| Diet habit | 136 | 1.115 | (0.519,2.392) | 0.781 |
| Parents origin | 142 | 0.950 | (0.440,2.048) | 0.895 |
| Parents education | 142 | 0.900 | (0.410,1.978) | 0.793 |
| Bottle feeding | 142 | 0.986 | (0.460,2.113) | 0.971 |
| Night meals | 135 | 1.091 | (0.506,2.353) | 0.824 |
| Sugar water | 137 | 0.822 | (0.372,1.819) | 0.629 |
| Sex | 142 | 0.979 | (0.458,2.094) | 0.956 |
| Heart problem | 142 | 0.988 | (0.462,2.114) | 0.975 |
| Cyanosis | 142 | 1.001 | (0.469,2.135) | 0.999 |
| Birth weight | 136 | 0.922 | (0.438,2.039) | 0.839 |
| Heart medication | 142 | 1.120 | (0.495,2.536) | 0.786 |
| Syndrome | 142 | 0.944 | (0.423,2.012) | 0.884 |
