# Supplementary material for: Effectiveness of an oral health intervention program for children with congenital heart defects
Source: BMC Oral Health. 2018 Mar 23;18:50. doi: 10.1186/s12903-018-0495-5 (PMC5865357; doi:10.1186/s12903-018-0495-5)
Supplement: Supplementary file 2 — Table S2. containing the outcome variable dental erosion and independent background factors for logistic regression model comparing the intervention with the control group. (DOCX 16 kb) [file 12903_2018_495_MOESM2_ESM.docx]

Table S2: Odds ratio (OR) and 95% confidence interval (CI) and p-values for outcome variable dental erosion and independent background factors for logistic regression model in the intervention compared to the control group.

|  | N | OR | CI | *p-value* |
| --- | --- | --- | --- | --- |
| Unadjusted | 133 | 1.167 | (0.503,2.705) | 0.719 |
| Brushing habit | 127 | 0.852 | (0.343,2.118) | 0.731 |
| Start age of tooth-brushing | 132 | 1.194 | (0.510,2.796) | 0.683 |
| Diet habit | 127 | 1.019 | (0.423,2.455) | 0.966 |
| Parents origin | 133 | 1.143 | (0.491,2.660) | 0.757 |
| Parents education | 133 | 1.168 | (0.503,2.711) | 0.718 |
| Bottle feeding | 133 | 1.097 | (0.468,2.572) | 0.832 |
| Night meals | 126 | 0.945 | (0.390,2.290) | 0.901 |
| Sugar water | 129 | 0.967 | (0.401,2.333) | 0.941 |
| Sex | 133 | 1.202 | (0.516,2.804) | 0.670 |
| Heart problem | 133 | 1.202 | (0.516,2.802) | 0.670 |
| Cyanosis | 133 | 1.164 | (0.502,2.700) | 0.724 |
| Birth weight | 127 | 1.021 | (0.420,2.479) | 0.964 |
| Heart medication | 133 | 1.481 | (0.591,3.710) | 0.402 |
| Syndrome | 133 | 0.958 | (0.394,2.328) | 0.924 |
