# Supplementary material for: Effectiveness of an oral health intervention program for children with congenital heart defects
Source: BMC Oral Health. 2018 Mar 23;18:50. doi: 10.1186/s12903-018-0495-5 (PMC5865357; doi:10.1186/s12903-018-0495-5)
Supplement: Supplementary file 3 — Table S3. with outcome variable dental plaque and independent background factors for logistic regression model comparing the intervention with the control group. (DOCX 15 kb) [file 12903_2018_495_MOESM3_ESM.docx]

Table S3: Odds ratio (OR) and 95% confidence interval (CI) and p-values for outcome variable dental plaque and independent background factors for logistic regression model in the intervention compared to the control group.

|  | N | OR | CI | *p-value* |
| --- | --- | --- | --- | --- |
| Unadjusted | 139 | 0.170 | (0.068,0.423) | <0.001 |
| Brushing habit | 133 | 0.194 | (0.074,0.504) | 0.001 |
| Start age of tooth-brushing | 138 | 0.166 | (0.066,0.419) | <0.001 |
| Diet habit | 133 | 0.142 | (0.055,0.367) | <0.001 |
| Parents origin | 139 | 0.167 | (0.067,0.418) | <0.001 |
| Parents education | 139 | 0.168 | (0.067,0.420) | <0.001 |
| Bottle feeding | 139 | 0.166 | (0.066,0.417) | <0.001 |
| Night meals | 132 | 0.163 | (0.065,0.411) | <0.001 |
| Sugar water | 134 | 0.166 | (0.062,0.442) | <0.001 |
| Sex | 139 | 0.171 | (0.069,0.426) | <0.001 |
| Heart problem | 139 | 0.170 | (0.067,0.420) | <0.001 |
| Cyanosis | 139 | 0.167 | (0.067,0.418) | <0.001 |
| Birth weight | 133 | 0.155 | (0.059,0.410) | <0.001 |
| Heart medication | 139 | 0.165 | (0.058,0.474) | 0.001 |
| Syndrome | 139 | 0.179 | (0.072,0.448) | <0.001 |
