# Supplementary material for: Effectiveness of an oral health intervention program for children with congenital heart defects
Source: BMC Oral Health. 2018 Mar 23;18:50. doi: 10.1186/s12903-018-0495-5 (PMC5865357; doi:10.1186/s12903-018-0495-5)
Supplement: Supplementary file 4 — Table S4. with outcome variable gingival bleeding and independent background factors for logistic regression model comparing the intervention with the control group. (DOCX 15 kb) [file 12903_2018_495_MOESM4_ESM.docx]

Table S4: Odds ratio (OR) and 95% confidence interval (CI) and p-values for outcome variable gingival bleeding and independent background factors for logistic regression model in the intervention compared to the control group.

|  | N | OR | CI | *p-value* |
| --- | --- | --- | --- | --- |
| Unadjusted | 132 | 0.163 | (0.044,0.603) | 0.007 |
| Brushing habit | 126 | 0.151 | (0.039,0.584) | 0.006 |
| Start age of tooth-brushing | 131 | 0.156 | (0.042,0.583) | 0.006 |
| Diet habit | 126 | 0.182 | (0.049,0.677) | 0.011 |
| Parents origin | 132 | 0.134 | (0.034,0.531) | 0.004 |
| Parents education | 132 | 0.157 | (0.042,0.584) | 0.006 |
| Bottle feeding | 132 | 0.167 | (0.045,0.621) | 0.008 |
| Night meals | 125 | 0.169 | (0.045,0.637) | 0.009 |
| Sugar water | 128 | 0.168 | (0.045,0.637) | 0.009 |
| Sex | 132 | 0.162 | (0.044,0.600) | 0.006 |
| Heart problem | 132 | 0.165 | (0.044,0.611) | 0.007 |
| Cyanosis | 132 | 0.157 | (0.042,0.586) | 0.006 |
| Birth weight | 127 | 0.178 | (0.046,0.682) | 0.012 |
| Heart medication | 132 | 0.148 | (0.031,0.712) | 0.017 |
| Syndrome | 132 | 0.173 | (0.046,0.651) | 0.009 |
